# Automated assessment of Ki‐67 proliferation index in neuroendocrine tumors by deep learning

**DOI:** 10.1111/apm.13190

**Published:** 2021-11-22

**Authors:** Tiina Vesterinen, Jenni Säilä, Sami Blom, Mirkka Pennanen, Helena Leijon, Johanna Arola

**Affiliations:** ^1^ Department of Pathology HUS Diagnostic Center HUSLAB University of Helsinki and Helsinki University Hospital Helsinki Finland; ^2^ Institute for Molecular Medicine Finland (FIMM) HiLIFE University of Helsinki Helsinki Finland; ^3^ Aiforia Technologies Oy Helsinki Finland

**Keywords:** deep learning, digital pathology, Ki‐67 proliferation index, neuroendocrine neoplasm

## Abstract

The Ki‐67 proliferation index (PI) is a prognostic factor in neuroendocrine tumors (NETs) and defines tumor grade. Analysis of Ki‐67 PI requires calculation of Ki‐67‐positive and Ki‐67‐negative tumor cells, which is highly subjective. To overcome this, we developed a deep learning‐based Ki‐67 PI algorithm (KAI) that objectively calculates Ki‐67 PI. Our study material consisted of NETs divided into training (*n* = 39), testing (*n* = 124), and validation (*n* = 60) series. All slides were digitized and processed in the Aiforia^®^ Create (Aiforia Technologies, Helsinki, Finland) platform. The ICC between the pathologists and the KAI was 0.89. In 46% of the tumors, the Ki‐67 PIs calculated by the pathologists and the KAI were the same. In 12% of the tumors, the Ki‐67 PI calculated by the KAI was 1% lower and in 42% of the tumors on average 3% higher. The DL‐based Ki‐67 PI algorithm yields results similar to human observers. While the algorithm cannot replace the pathologist, it can assist in the laborious Ki‐67 PI assessment of NETs. In the future, this approach could be useful in, for example, multi‐center clinical trials where objective estimation of Ki‐67 PI is crucial.

## INTRODUCTION

Neuroendocrine neoplasms (NENs) arise from cells of the diffuse neuroendocrine system dispersed throughout the body. NENs are a rare tumor entity comprising approximately 2% of all malignancies [1]. The most common primary tumor locations are the gastrointestinal tract (65%) and lungs (25%) [2]. While NENs share neuroendocrine differentiation based on immunolabeling, for example, chromogranin A and synaptophysin, they present widely differing etiologies, morphological and genomic findings, hormonal activity, clinical presentation, and prognosis.

NENs are in general classified by morphological features and immunohistochemical biomarkers as well‐differentiated neuroendocrine tumors (NETs) and poorly differentiated neuroendocrine carcinomas (NECs) [3]. All NECs are high‐grade malignancies with >20 mitoses per 2 mm^2^ and a Ki‐67 proliferation index (PI) of >20%. Most NETs present low (1‐20%) proliferation and are graded as G1 or G2 based on mitoses per 2 mm^2^ and a Ki‐67 PI. G3 tumors have a Ki‐67 PI >20%. Of note, Ki‐67 PI assessment is not included in the current WHO classification for pulmonary NENs [4]. However, in both gastroenteropancreatic (GEP)‐NETs and pulmonary NETs, Ki‐67 PI is considered as a prognostic factor [5, 6].

Ki‐67 PI assessment starts with immunohistochemical labeling of tumor cells with a validated primary antibody typically clone MIB‐1. After this, the percentage of tumor cells expressing Ki‐67 is determined by counting at least 500 tumor cells or 0.4 mm^2^ of the tumor area in the highest labeling regions (hotspots) [3, 7]. Several scoring methods are available including eyeball estimation, manual counting of cells (through microscope eyepiece, using a printed image or from a monitor), and computer‐assisted quantification using digital image analysis [8, 9, 10, 11, 12, 13]. However, to our knowledge, no consensus exists on the best method. Eyeball estimation has shown to suffer from considerable interobserver variation and is thus discouraged [11, 12, 13]. More reproducible manual counting method is accurate but has typically a long application time and is labor‐intensive [8, 11, 12]. To eliminate manual counting, automated counting methods for Ki‐67 PI utilizing digital images have been developed for clinical practice. Many of them have been evaluated in NETs with fluctuating results: Others state that digital image analysis yields similar results than manual counting while others experience miscalculations [9, 10, 11, 12, 14, 15, 16, 17, 18].

The development of machine learning techniques has opened new avenues in histopathology [19]. Deep learning (DL) is a subtype of machine learning in which algorithms are trained for specific tasks by exposing a multilayered artificial neural network to training data [19]. In the case of supervised learning, the algorithm is trained with human‐made training annotations [20]. In histopathology, this means that a human being annotates desired features by labeling digital tissue images. This creates the ground truth, the reference from which the neural network learns.

Several DL‐based algorithms have been developed but very few of them have reached clinical implementation [19]. In the field of NETs and Ki‐67 PI, researchers have utilized DL for example to improve accuracy and save time in Ki‐67 PI analysis by using Ki‐67 and synaptophysin double‐immunostained slides or by predicting Ki‐67‐positive cells directly from hematoxylin and eosin‐stained slide [21, 22]. Both methods are novel but not easily implemented in clinical routine.

Here, we aimed to train a DL‐based algorithm for automated assessment of Ki‐67 PI in NETs and compared its performance to human observers. To our knowledge, this approach is now documented for the first time for NETs.

## MATERIALS AND METHODS

This study consisted of two steps: 1) development and testing of a DL‐based algorithm for calculating Ki‐67 PI in NETs and 2) validation of the algorithm in an independent slide series. In addition, variation in Ki‐67 PI analysis between human observers was evaluated.

### Tumor specimens

Three tumor series were used: DL training, DL testing, and DL validation series (Table 1). Pulmonary NETs for training were a part of our previous study where the material was collected from the Finnish Biobanks [23]. Pancreatic NETs for training were collected from the archives of the Department of Pathology, Helsinki University Hospital (HUH), Helsinki, Finland.

**Table 1:** Tumor series for training, testing, and validation of the Ki‐67 proliferation index algorithm

	Training series	Testing series	Validation series	
			Gradus 1	Gradus 2
PNET	25		2	18
SI‐NET			7	13
PC	14	124	20	

PC, pulmonary carcinoid; PNET, pancreatic neuroendocrine tumor; SI‐NET, small‐intestinal neuroendocrine tumor.

The DL testing series comprised 124 previously reported pulmonary NETs, none of which was included in the DL training series [24]. The DL validation series included whole slides of 60 NETs, none of which was involved in the DL training or in the DL testing series. These tumors were surgically removed between 2015 and 2019 at HUH, formalin‐fixed and paraffin‐embedded. The original Ki‐67‐labeled slides were retrieved from the archives of the Department of Pathology, HUH and digitized. The study protocol was approved by Ethics Committee IV of HUH (HUS/1258/2020). Informed consents were not obtained since the study utilized only slides.

### Immunohistochemistry and whole‐slide imaging

Immunohistochemical labeling for Ki‐67 was performed at the Department of Pathology, HUH. Briefly, 3.5 µm sections were cut on adhesive slides and deparaffinized. Antigen retrieval was performed using CC1 reagent (Ventana Medical System, Inc., Roche, Tucson, AZ, USA), and the primary antibody Ki‐67 (clone MIB‐1, dilution 1:100, Dako, Agilent Pathology Solutions, Santa Clara, CA, USA) was incubated for 32 min. Immunoreactions were visualized with OptiView Universal DAB Detection Kit (Ventana Medical System) and counterstained with hematoxylin.

Ki‐67‐labeled slides were digitized with a Pannoramic 250 FLASH III whole‐slide scanner (3DHISTECH, Budapest, Hungary) using a 20x objective with a resolution of 0.242 µm/pixel. The digitized images were uploaded to Aiforia^®^ (Aiforia Technologies, Helsinki, Finland), which is a commercial cloud‐based platform for managing and viewing digitized whole‐slide images and for training neural networks for automated image analysis.

### Training and testing of the Ki‐67 PI algorithm

**Fig. 1:**
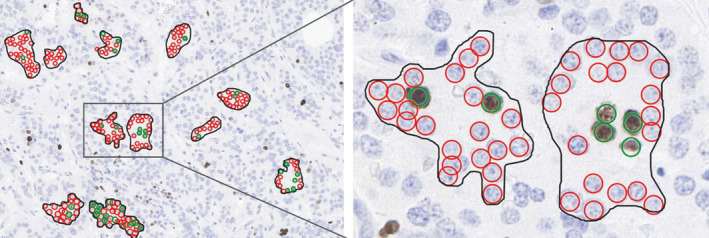
Training of the Ki‐67 PI algorithm by manually annotating Ki‐67^pos^ and Ki‐67^neg^ pulmonary NET cells. Green circle indicates a Ki‐67^pos^ tumor cell and red circle a Ki‐67^neg^ tumor cell. Training areas are surrounded by a black line. Several small training areas were drawn, and all cells within them were annotated.

In Aiforia^®^ Create, we first trained a deep convolutional neural network algorithm to identify the tissue on the slides and then recognize Ki‐67^pos^ and Ki‐67^neg^ tumor cells as objects in 14 pulmonary NET samples. The training data for our Ki‐67 PI algorithm (KAI) included supervised manual annotations of 354 Ki‐67^pos^ and 3003 Ki‐67^neg^ pulmonary NET cells (Fig. 1). Since we aimed to classify tumor cell nuclei rather than whole cells, we used an object feature size of 7 µm, which fitted inside the nuclei, together with the following augmentation of the training image data: size scaling between −20% and 20%, 20% aspect ratio change, 20% shear distortion, luminance change between −20% and 20%, contrast change between −20% and 20%, 5% white balance change, noise level of 5 units, jpg compression quality between 40% and 60% in 0.5% of the training data per training epoch, and blurring using a blur radius of one pixel in 0.5% of the training data per training epoch.

To evaluate the accuracy of the KAI, we compared its output with the ground truth on a DL testing series of 124 pulmonary NETs. After successful testing, we introduced 25 pancreatic NETs to the KAI by manually annotating 327 Ki‐67^pos^ and 1301 Ki‐67^neg^ pancreatic NET cells.

### Validation of the KAI

The performance of the KAI was validated against manual Ki‐67 PI estimation by three independent observers (J.A., H.L., and M.P.) in 20 pancreatic NETs, 20 small‐intestinal NETs, and 20 pulmonary NETs independent of the DL training or DL testing series. All three human observers were specialized endocrine pathologists familiar with scoring Ki‐67 PI, and they interpreted three pre‐marked hotspot regions per tumor from their own computer screens by manually calculating the positive tumor cell percentage. To calculate one Ki‐67 PI value per tumor, three scores per tumor were averaged.

To prepare the KAI for validation, we used one randomly selected slide of each NET type from the DL validation series to introduce the algorithm technical variation (e.g., thickness of section and intensity of staining). These slides were excluded from the validation. Altogether, 109 Ki‐67^pos^ and 1719 Ki‐67^neg^ NET cells were used in the preparing. After this, the same regions interpreted by the three pathologists were analyzed with the KAI. Similarly to manual analysis, results from three hotspots per tumor were averaged.

### Statistical analyses

Manual training annotations and the detection made by the KAI were compared in the Aiforia^®^ Create platform. False positive (FP) refers to the objects that were not annotated, but were detected by the KAI (independent of the object class, that is, Ki‐67 positive or negative). False negative (FN) refers to the objects that were annotated, but were not detected by the KAI, again independent of the object class. The percentage of FP and FN was calculated by dividing the count of FP and FN by the total count of annotated objects. Total area error was the sum of FP and FN.

The level of agreement between the pathologists and the KAI was tested by the intraclass correlation coefficient (ICC) using the model 3 “two‐way mixed,” form 1 “single measures,” and type “absolute agreement” [25]. Values of <0.5, 0.5–0.75, 0.75–0.90, and >0.90 indicate poor, moderate, good, and excellent reliability, respectively. Bland–Altman plot was drawn to graphically display the differences between the two scoring methods [26]. Statistical analysis was carried out by using the Statistical Package for Social Sciences software version 25.0 (SPSS; Chicago, IL, USA).

## RESULTS

### Testing of the KAI with pulmonary NETs

After the first round of trainings, the KAI showed a total object error of 3.78% (false positive 1.58% and false negative 2.20%). The ICC to measure agreement between the KAI and the previously reported ground truth in the DL testing series was 0.90 (95% CI 0.85–0.94).

### Agreement between the pathologists scoring the Ki‐67 PI manually

**Fig. 2:**
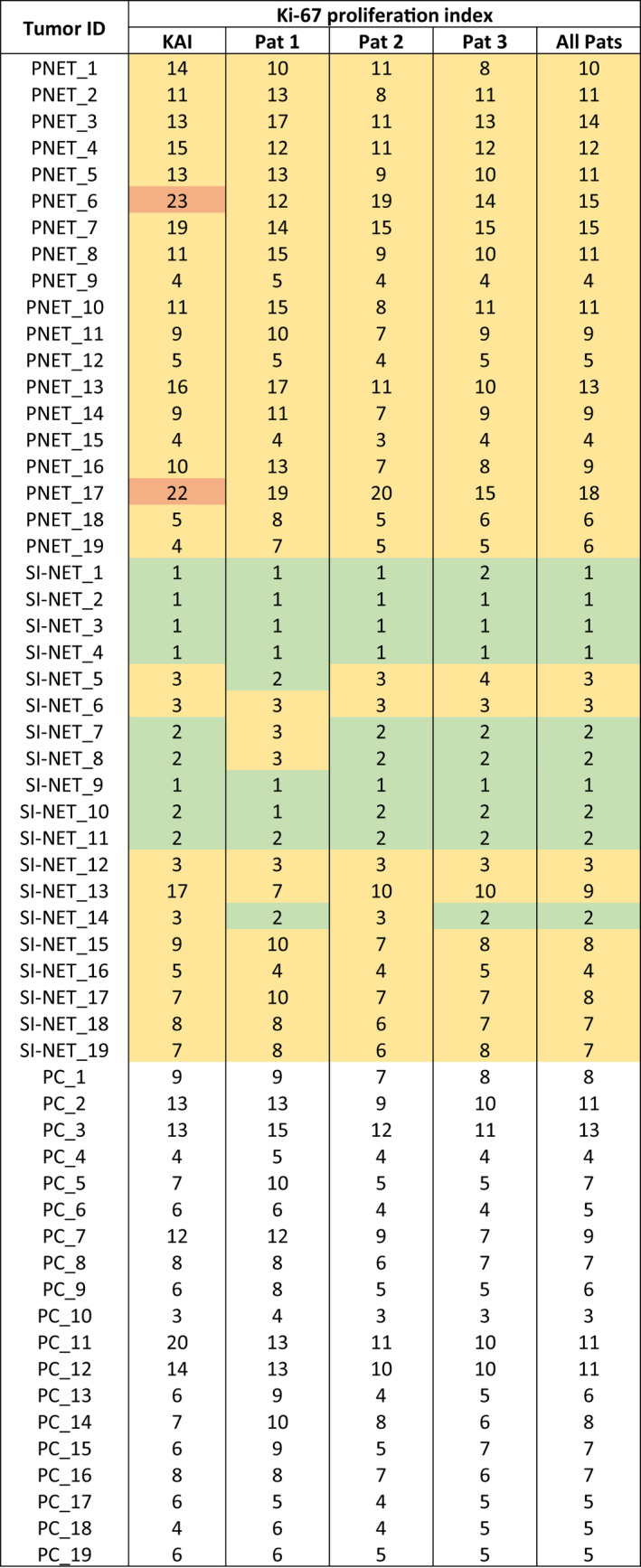
Heat map of Ki‐67 PI scores. Rows represent samples, and columns represent scorers (KAI; Ki‐67 PI algorithm, Pat = pathologist). All values are averaged values per three hotspot areas. Green color indicates Ki‐67 PI <3% (grade 1), yellow 3‐20% (grade 2), and orange >20% (grade 3) for pancreatic neuroendocrine tumors (PNETs) and small‐intestinal neuroendocrine tumors (SI‐NETs). For pulmonary carcinoid tumors (PCs), the gradus is not given since it is not a part of their classification.

In the DL validation series, all three pathologists interpreted three hotspot regions per tumor containing on average 225 tumor cells per hotspot (median 223, range 182–279, as calculated with the KAI). The ICC to measure agreement among the pathologists in the Ki‐67 PI scoring was 0.84 (95% CI 0.66–0.91). ICCs to measure pairwise agreement between the pathologists are shown in Table 2. Fig. 2 presents the Ki‐67 PI scores in a heat map format categorized by NET grades (Ki‐67 PI <3% = G1, 3–20% = G2 or >20% = G3, no mitoses taking into account).

**Table 2:** Intraclass correlation coefficient agreement between the pathologists and the Ki‐67 PI algorithm (KAI)

	Pathologist 2	Pathologist 3	KAI
Pathologist 1	0.78 (95% CI 0.39–0.90)	0.82 (95% CI 0.46–0.93)	0.86 (95% CI 0.77–0.92)
Pathologist 2		0.94 (95% CI 0.85–0.97)	0.87 (95% CI 0.49–0.95)
Pathologist 3			0.83 (95% CI 0.62–0.91)

CI, confidence interval; PI, proliferation index.

### Agreement between the pathologists and the KAI in the Ki‐67 PI analysis

After training the KAI with pancreatic NETs and preparing it for the validation, the total object error was 2.75% (false positive 0.93% and false negative 1.82%). To test the accuracy of the KAI, the exact same hotspot regions that had been scored by the pathologists were analyzed with the KAI, and the generated Ki‐67 PI values were compared with the pathologists’ results. The ICC between the pathologists and the KAI was 0.84 (95% CI 0.74–0.91). When the Ki‐67 PI scorings were averaged among the three pathologists and compared with the KAI, the ICC value was 0.89 (95% CI 0.78–0.94). ICCs to measure pairwise agreement between the pathologists and the KAI are shown in Table 2. The Ki‐67 PI scores in a heat map format are shown in Fig. 2.

**Fig. 3:**
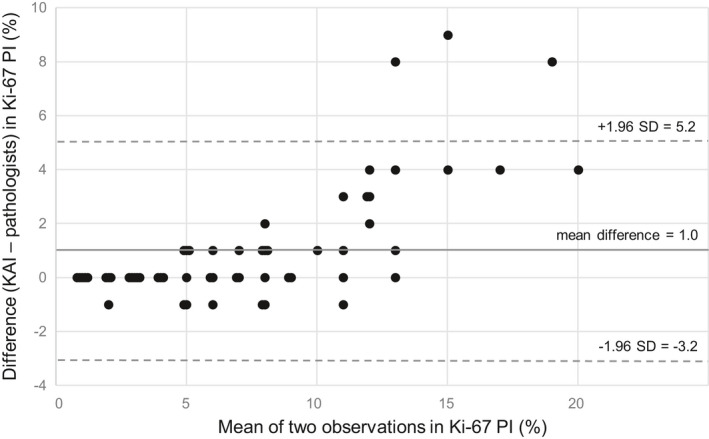
Bland–Altman plot for Ki‐67 PI observed by three pathologists (averaged value) or the Ki‐67 PI algorithm (KAI). SD, standard deviation.

**Fig. 4:**
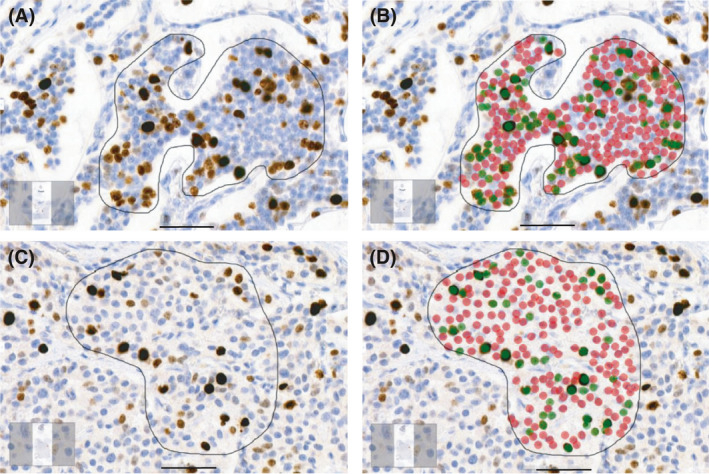
Analysis of hotspot regions with the Ki‐67 PI algorithm (KAI). The KAI marks Ki‐67^neg^ tumor cells with red and Ki‐67^pos^ tumor cells with green and calculates the cell numbers and percentages. Analysis areas are confined with a black line. (A) Example of a pancreatic NET hotspot region where scanning was not in focus and cells were overlapping. (B) The same area as in A, with the KAI marking Ki‐67^pos^ and Ki‐67^neg^ tumor cells. (C) Example of a small‐intestinal NET hotspot region where the KAI calculated cell numbers correctly (D), but the pathologists overestimated the number of negative cells. Images taken with magnification 40x; scale bar 50 µm.

Bland–Altman plot was drawn to compare the results of two analysis methods based on the mean values and the differences of the Ki‐67 PI for each case (Fig 3). The Ki‐67 PI calculated by the KAI was compared to the averaged Ki‐67 PI of the three pathologists. In the plot, the mean difference of the methods was 1% and the limits of agreement values were 5.2% and −3.2%. In 46% (26/57) of the tumors, there was no difference in Ki‐67 PI between the averaged value of the three pathologists and the KAI. In seven tumors (12%), the Ki‐67 PI calculated by the KAI was 1% lower. In the rest 24 tumors (42%), the KAI showed higher Ki‐67 PI with an average of 3% (median 2%, range 1‐9%). Two of these tumors were attributed to mild focusing problems in scanning and showed overlapping cells (Fig. 4).

### Agreement between the pathologists and the KAI in grading tumors

When considering only Ki‐67 PI, not the number of mitoses, the pathologists and the KAI unanimously set tumor grades for 17 of the 19 pancreatic NETs (90%). In two discrepant cases, Ki‐67 PI value analyzed with the KAI was 22% or 23% indicating grade 3, whereas pathologists scored the Ki‐67 to be <20%, thus assigning grade 2 (Table 3). In small‐intestinal NETs, the pathologists and the KAI agreed on tumor grades in 15 of the 19 cases (79%). Four discrepant cases are described in Table 3.

**Table 3:** Discrepant cases in terms of grading based on the Ki‐67 proliferation index

	Pathologist 1		Pathologist 2		Pathologist 3		Ki‐67 PI algorithm	
	Ki‐67 PI	Grade	Ki‐67 PI	Grade	Ki‐67 PI	Grade	Ki‐67 PI	Grade
PNET_6	12	G2	19	G2	14	G2	23	G3
PNET_17	19	G2	20	G2	15	G2	22	G3
SI‐NET_5	3	G2	3	G2	3	G2	2	G1
SI‐NET_7	2	G1	3	G2	4	G2	3	G2
SI‐NET_8	3	G2	2	G1	2	G1	2	G1
SI‐NET_14	2	G1	3	G2	2	G1	3	G2

PI, proliferation index; PNET, pancreatic neuroendocrine tumor; SI‐NET, small‐intestinal neuroendocrine tumor.

## DISCUSSION

Here, we presented how deep learning (DL) can be implemented in the Ki‐67 PI assessment of NETs. Our results show that DL‐based algorithm can assist pathologist in calculating Ki‐67 PI but it cannot replace pathologist in its current form. However, the agreement between the algorithm and three endocrine pathologists, as measured with ICC, is similar to the agreement between the three pathologists.

Gastroenteropancreatic neuroendocrine tumors (GEP‐NENs) represent a heterogeneous group of tumors featuring differences in prognosis. In the current WHO classification scheme, mitotic count and Ki‐67 PI are decisive parameters when grading the tumors [7]. Tumor grade, in turn, has an impact on the treatment of the patients; thus, standardized Ki‐67 PI scoring is essential. Typically, Ki‐67 PI in NECs is over 50% and thus easier to estimate than a cutoff of 3% or 20% for G1, G2, and G3 NET. This is why we included G1 and G2 tumors in our study. G3 tumors were missing from our cohort since WHO did not introduce this category until 2019 [7].

Currently, consensus regarding the best method to assess the Ki‐67 PI in NENs is lacking. Eyeball estimation, manual counting, and digital image analysis are the three main methods for scoring [8, 9, 10, 11, 12, 13]. Eyeballing is not encouraged due to its low reproducibility and high inter‐reader variability [11, 12, 13]. The manual method involves printing of an image and marking of Ki‐67‐positive and Ki‐67‐negative tumor cells, which is impractical, time‐consuming, and labor‐intensive, although accurate [11]. Despite inter‐ and intra‐observer variability, both methods are still preferred over digital image analysis in many clinical pathology departments due to their minimal disruption of the current workflow not pertaining digital pathology. While manual counting of Ki‐67‐positive and Ki‐67‐negative tumor cells on a digital image was shown to have near‐perfect agreement with manual counting on a printout image, the technique itself is still labor‐intensive [8].

Current North American Neuroendocrine Tumor Society (NANETS) consensus guidelines recommend manual counting of camera‐captured digital images over eyeballing when calculating Ki‐67 PI for pancreatic NETs [5]. NANETS also approves digital image analysis if it is locally validated. In fact, to overcome the interobserver variability and cumbersomeness present in manual counting, automated counting methods are preferred [9, 10, 12, 18]. In recent years, several studies have attempted to establish an automated calculation method for Ki‐67 PI in NETs. Different commercial solutions like Ventana Virtuoso (Roche Diagnostics, Rotkreuz, Switzerland), Aperio Nuclear Algorithm (Leica Biosystems Inc., IL, USA), Automated Cellular Imaging System (ACIS) (Dako, Carpinteria, CA), 3DHistech QuantCenter (3DHistech, Budapest, Hungary), and HALO image analysis platform (Indica Laboratories, NM, USA) have been studied with typically promising results [9, 10, 11, 12, 14, 15, 18 ]. However, researchers have experienced inability of the software to distinguish Ki‐67‐positive tumor cells from other Ki‐67‐labeling cell types like lymphocytes, endothelial cells, and stromal cells, cells with abundant background pigment (e.g., hemosiderin) or to detect all negative tumor nuclei [11, 15]. Similarly, open‐source software or free web application such as QuPath and ImmunoRatio have been studied in calculating Ki‐67 PI in NETs [16, 27]. Owens et al. reported QuPath to over‐estimate Ki‐67 PI due to a tendency to ascribe positivity to tumor cells that were interpreted as negative by a pathologist [27]. The main reasons for this phenomenon were the presence of increased section thickness, nuclear crowding, or excess background staining.

We experienced similar difficulties with our algorithm (KAI). The KAI showed the same Ki‐67 PI value as pathologists in 46% of the tumors, one percent lower Ki‐67 in 12% of tumors but on average 3% higher Ki‐67 PI in 42% of the tumors. These tumors were attributed to mild focusing problems in scanning and showed overlapping cells, which caused the KAI to detect less Ki‐67‐negative tumor cells than the pathologists did. In addition, the KAI marked some of only faintly labeled tumor cells as Ki‐67 positive. These factors led to a higher Ki‐67 PI than in the analysis performed by the pathologists. In some cases, the pathologists detected less negative tumor cells than the KAI did, which led to a higher Ki‐67 PI assigned by the pathologists. On the contrary, the KAI did not detect non‐tumoral Ki‐67 labeled cells or cells with background pigment since it was only trained to detect tumor cells.

One critical aspect in training an algorithm to detect Ki‐67‐positive and Ki‐67‐negative cells is defining the positivity and negativity, which is to some extent subjective. We defined positive tumor cells as tumor cells presenting moderate to strong brown stain in the nucleus and no counterstain at all. In this sense, KAI was trained to interpret pale brown tumor nuclei as negative. Several attempts to increase concordance in Ki‐67 scoring have been made especially in breast cancer diagnostics, where Ki‐67 PI is essential. For instance, Polley et al. presented a web‐based tool (http://www.gpec.ubc.ca/calibrator) to calibrate pathologists to Ki‐67 scoring [28]. In our case, agreement between the pathologists was good, which was probably due to their being from the same institute and working closely for many years. Thus, the criteria for positive and negative nuclear staining were similar. More variance in Ki‐67 PI values would be expected if more pathologists from different departments were involved. However, this variance can be decreased by external quality assessment schemes and regular participation in these proficiency programs [29].

Despite drawbacks, computer‐assisted analyses are suggested to be more reproducible, offer high‐capacity analysis, and eliminate human errors [9, 12, 14]. These methods also have the potential to reduce pathologists’ workload, which in the growing shortage of pathologists worldwide is essential [30, 31]. Nevertheless, it should be recognized that the implementation of digital image analysis requires substantial input of both pathologists and technologists. In addition, pathologist supervision of image analysis software or algorithm is crucial when deployed in clinical practice. A strength of a DL algorithm is that it can be easily trained more to detect challenging features and it can adapt to what it has learned. For instance, in this study, we did not train the KAI with small‐intestinal NET cells, but it still recognized them.

In addition to challenges in choosing the best scoring method for Ki‐67 PI, several other issues need to be taken into account. Apart from variables in immunohistochemical staining, these include defining tumor borders and what constitutes a hotspot. NETs present usually high tumor cell percentage and lack cellular stroma component and tumor‐infiltrating lymphocytes typical, for example, adenocarcinoma, which simplifies Ki‐67 PI calculation in NETs [7]. Still, approaches like synaptophysin‐Ki‐67 double‐stain may be helpful in eliminating false‐positive signals and in improving interobserver agreement [21, 32]. Moreover, recent advances in virtual double‐staining allow digital aligning of serial sections stained with Ki‐67 and, for example, cytokeratin as described for breast cancer diagnostics [33].

Intratumoral heterogeneity, which is a known feature of pancreatic NETs, as well as the subjectivity of hot spot selection, can lead to marked variation in Ki‐67 PI [27]. In this study, hotspot areas were marked in advance and the idea was to compare KAI’s performance with that of pathologists, not to find the best method for recognizing a hotspot. Naturally, the identification of hotspots is as irreproducible as counting and can be an application of DL in NETs as shown by Balkenhol et al. for breast cancer [34].

For statistical ICC analysis, we chose model 3 (two‐way mixed) instead of model 2 (two‐way random) for three reasons. First, the gold standard is subjective, and there is a factual disagreement between pathologists in general. Second, we were not able to reliably establish, whether our study pathologists and their performance were truly representative of the general population of pathologist, and third, there is no global ground truth for calculating Ki‐67 PI. Thus, we decided to establish a study‐specific gold standard (ground truth) and to test the concordance between study pathologists without seeking the results to generalize to all pathologists.

This study has strengths and limitations. The main strength is that we utilized original Ki‐67 labeled slides from three different NETs for validation of the KAI. In addition, three endocrine pathologists calculated Ki‐67 PIs manually, and we could compare their agreement in scoring. Limitations include the fact that the pathologist did not use the same computer monitor in scoring, which might affect in calculating of faintly stained tumor cells. Moreover, we lack the results of manual scoring of printed images. However, with these promising results, we aim to utilize this algorithm in outcome studies of NET patients as well as try to validate the algorithm with a larger, external tumor series before implementation into clinical practice.

## CONCLUSION

In summary, the Ki‐67 PI is a critical parameter in grading NETs and determining patients’ treatment and prognosis. DL‐based image analysis algorithm can assist pathologists in determining Ki‐67 PI more accurately and objectively if implemented into clinical practice, but it cannot replace the pathologist. In the future, accurate and reproducible Ki‐67 PI values, alone or coupled with other parameters, might offer a tool for classifying NETs into several groups with regard to prognosis, similarly to adrenocortical tumors [35]. In addition, this web‐based approach could be useful in, for example, multi‐center clinical trials where objective estimation of Ki‐67 PI is crucial.

## FUNDING

This work was supported by the Cancer Foundation Finland [no grant number] and the Helsinki University Hospital Research Fund [grant number TYH2019205].

## CONFLICT OF INTEREST

Sami Blom reports being an employee of Aiforia Technologies Oy. Other authors have no conflicts of interest to declare that are relevant to the content of this article.

## AUTHOR CONTRIBUTIONS

Jenni Säilä and Sami Blom trained the algorithm. Johanna Arola, Helena Leijon, and Mirkka Pennanen manually scored Ki‐67 PIs. Tiina Vesterinen analyzed the data, created tables and figures, and wrote the first draft of the manuscript. All authors contributed to the study conception and design, commented on previous versions of the manuscript and approved the final manuscript.

## ETHICS APPROVAL

The study protocol was approved by Ethics Committee IV of HUH (HUS/1258/2020). Based on the Finnish legislation, no informed consent is needed for studies, which involve no personal data.
